# Supplementary material for: Production and Immunogenicity of a Tag-Free Recombinant Chimera Based on PfMSP-1 and PfMSP-3 Using Alhydrogel and Dipeptide-Based Hydrogels
Source: Vaccines (Basel). 2021 Jul 13;9(7):782. doi: 10.3390/vaccines9070782 (PMC8310097; doi:10.3390/vaccines9070782)
Supplement: Supplementary file 1 [file vaccines-09-00782-s001.zip › vaccines-1197260-supplementary.pdf]

Supplementary information:

Supplementary S1

Synthesis of Dipeptides, FΔF and LΔF:

Briefly, Boc-Phe-OH or Boc-Leu-OH (5 mM) was dissolved in dry THF, chilled to -20 °C, followed by stirring in an ice-salt bath. Next, equivalent moles of NMM and IBCF were added and left for 10 minutes. Pre-cooled aqueous solutions of DL-3-β-phenylserine (5.5 mM) and sodium hydroxide (5.5 mM) were then added, stirred overnight and concentrated in vacuo. The obtained residue was acidified with citric acid and extracted with ethyl acetate. The ethyl acetate layer was washed thrice with water and saturated sodium chloride. It was then dried over anhydrous sodium sulfate and evaporated to get Boc-Phe-DL-3-β-phenylserine or Boc-Leu-DL-3-β-phenylserine which was next mixed with anhydrous sodium acetate (6.5 mM) in freshly distilled acetic anhydride (50 ml) and stirred for 36 hours. The thick slurry thus obtained was poured over crushed ice. The precipitate was filtered, washed with 5% $NaHCO_3$, cold water, and dried under vacuum. The resulting compounds (Boc-Phe-ΔPhe-Azalactone or Boc-Leu-ΔPhe-Azalactone) were dissolved in methanol and treated with 1.5 equiv of 1 N NaOH solution for 3-4 hours. It was concentrated in vacuo. Acidification with solid citric acid liberated the peptide which was extracted in ethyl acetate, to yield Boc-Phe-ΔPhe-COOH or Boc-Leu-ΔPhe-COOH. Boc group was deprotected using anhydrous TFA and the free dipeptide was purified on a preparative reverse phase C-18 column, using acetonitrile-water linear gradient of 5-95% (1mL/min). The purified FΔF and LΔF were analysed by mass spectroscopy (Applied Biosystems QStar (Q-TOF), retention time 15 minutes. Observed mass, 311 Da (310 + 1H+); expected mass, 310 Da and 277.2 Da respectively.

Supplementary Figures

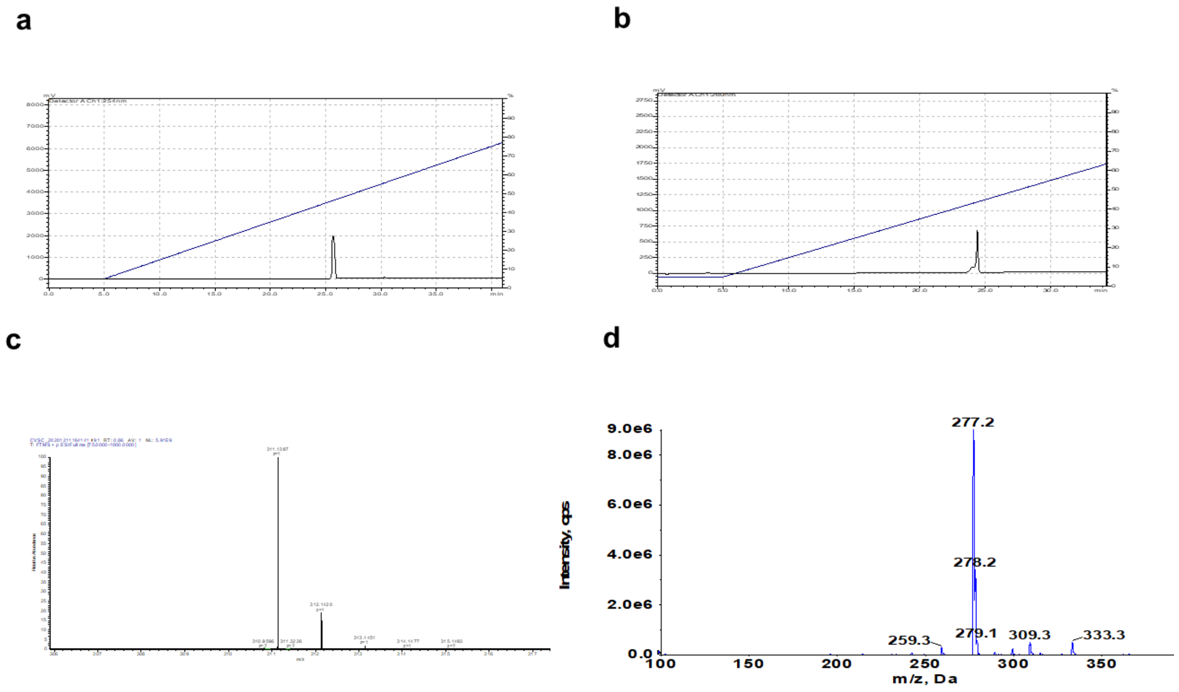

Figure S1. Characterization FΔF and LΔF. (a) & (b) reverse phase high performance liquid chromatography (RP-HPLC); (c) & (d) Intact mass spectrometry of FΔF and LΔF

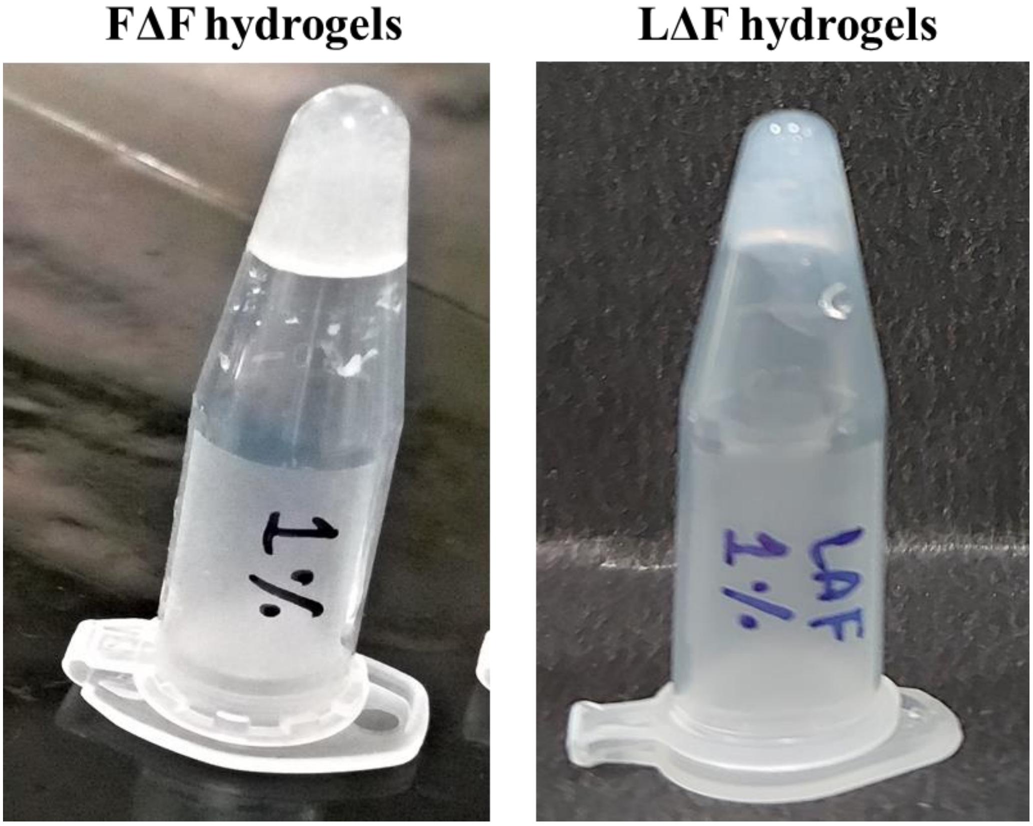

Figure S2. Formation of FΔF and LΔF hydrogels**.** 1 wt% FΔF and LΔF hydrogels formation in the presence of sodium acetate buffer (0.8M, pH 7) at room temperature.

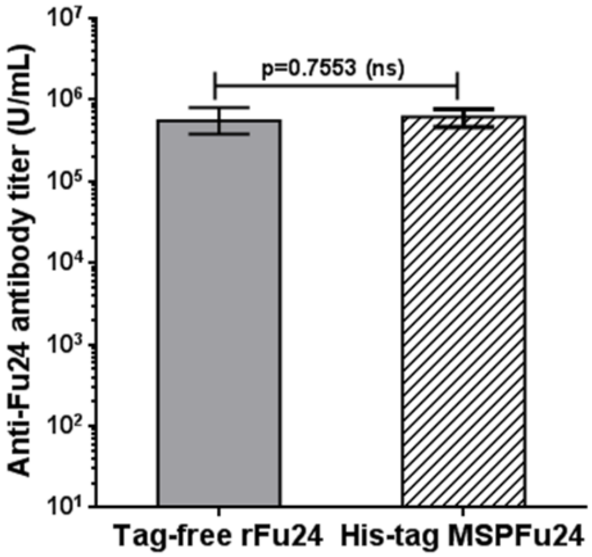

Figure S3. Comparison of antibody responses to tag-free and His-tagged fusion chimera proteins. Balb/c mice (n=6) were immunized at 4-weeks interval with 25ug/dose of tag-free 2Fu24 or his-tag rPfMSP-Fu24 formulated with Freund's adjuvant and Alhydrogel. Sera collected before and after the final immunization were analyzed for antigen-specific IgG titers by ELISA. The end point titres represented as means ± SEM. Statistical significance between groups was determined by t-test (ns: not significant).

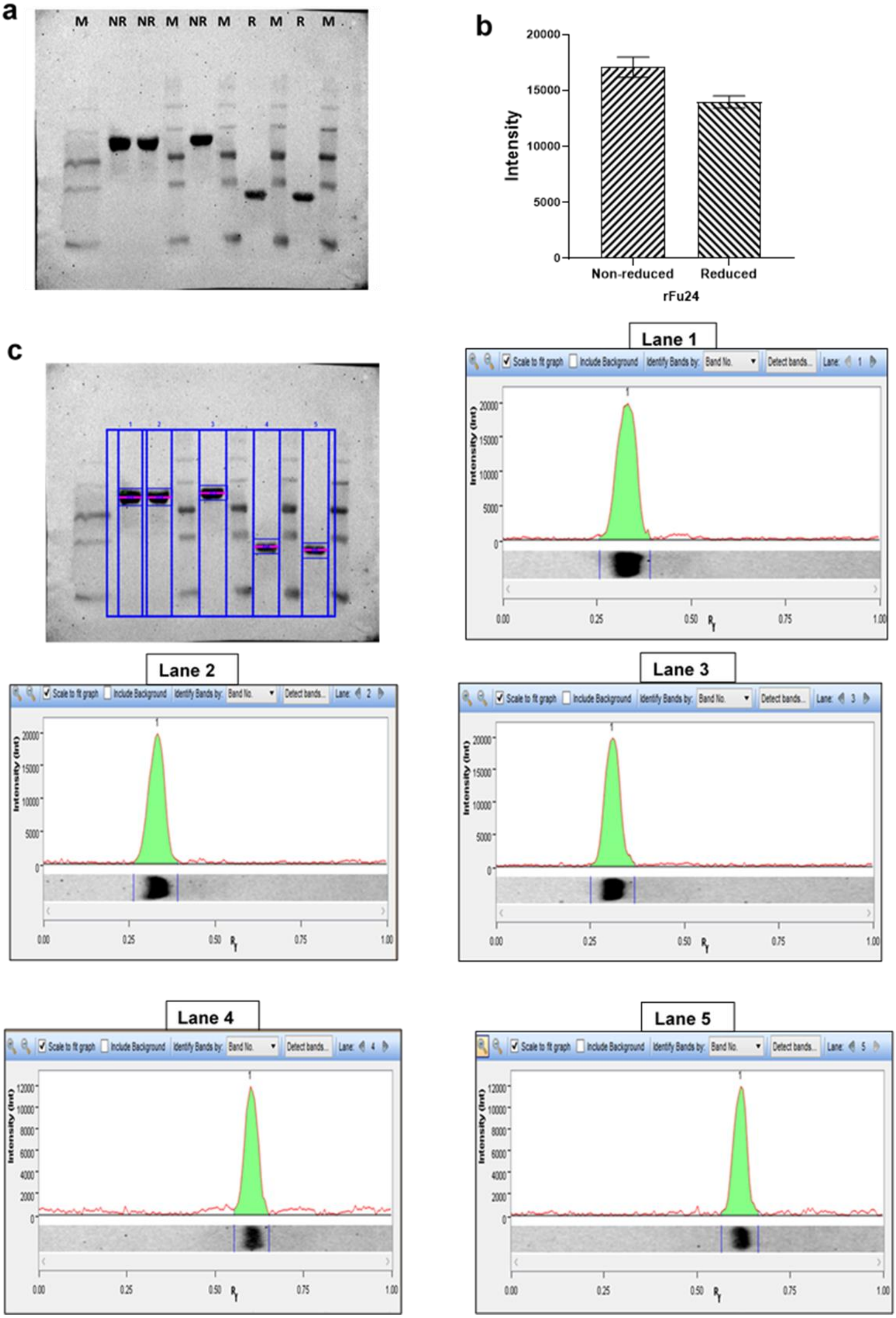
a
M NR NR M NR M R M R M
b
20000
15000
10000
5000
0
Intensity
Non-reduced
Reduced
rFu24
c
Lane 1
Lane 2
Lane 3
Lane 4
Lane 5
Scale to fit graph
Include Background
Identify Bands by:
Band No.
Detect bands...
Intensity (Int)
Rf

Figure S4. (a) Western blot of reduced and non-reduced rFu24 detected by anti-rFu24 polyclonal sera. (b) Intensity of the bands of reduced and non-reduced rFu24 detected by anti-rFu24 polyclonal sera and immunoblot bands were quantified using Image Lab^{TM} software, version 6.0.0 build 25, Bio-Rad Laboratories, Inc. USA, 2017. (c) Histogram plots of each band of western blot using Image Lab^{TM}, version 6.0.0 build 25, Bio-Rad Laboratories, Inc. USA, 2017.
